# The ptotic tongue—imaging appearance and pathology localization along the course of the hypoglossal nerve

**DOI:** 10.1007/s00234-023-03204-y

**Published:** 2023-08-04

**Authors:** Vineet Vijay Gorolay, Ngoc-Anh Tran, Ryan Tade, Kristen Baugnon, Ashley Aiken, Xin Wu

**Affiliations:** 1 Department of Radiology and Biomedical Imaging, University of California San Francisco, 505 Parnassus Ave, San Francisco, CA 94143 USA; 2 Radiology Associates, Springfield, OR USA; 3 Department of Radiology and Imaging Science, Emory University, Atlanta, GA USA

**Keywords:** MRI, Hypoglossal, Ptosis, Hypoglossal palsy, Denervation

## Abstract

CT and MRI findings of tongue ptosis and atrophy should alert radiologists to potential pathology along the course of the hypoglossal nerve (cranial nerve XII), a purely motor cranial nerve which supplies the intrinsic and extrinsic muscles of the tongue. While relatively specific for hypoglossal nerve pathology, these findings do not accurately localize the site or cause of denervation. A detailed understanding of the anatomic extent of the nerve, which crosses multiple anatomic spaces, is essential to identify possible underlying pathology, which ranges from benign postoperative changes to life-threatening medical emergencies. This review will describe key imaging findings of tongue denervation, segmental anatomy of the hypoglossal nerve, imaging optimization, and comprehensive imaging examples of diverse pathology which may affect the hypoglossal nerve. Armed with this knowledge, radiologists will increase their sensitivity for detection of pathology and provide clinically relevant differential diagnoses when faced with findings of tongue ptosis and denervation.

## Introduction

The hypoglossal nerve (cranial nerve XII) arises from the hypoglossal nucleus in the medulla and provides motor innervation to all the intrinsic and extrinsic muscles of the tongue, except the palatoglossus muscle which is innervated by the pharyngeal plexus of the vagus nerve (cranial nerve X). In its carotid and sublingual segments, the hypoglossal nerve sheath also carries fibers from the first cervical spinal nerve root/superior root of the ansa cervicalis, which provides motor innervation to the geniohyoid and thyrohyoid muscles. Injury produces characteristic clinical and imaging findings which include unilateral tongue ptosis and denervation atrophy. The ability to recognize the CT and MRI appearance of these findings is the first step to accurately identify hypoglossal nerve dysfunction.

Once hypoglossal nerve dysfunction is suspected, imaging should be tailored to image the entirety of the nerve from the medulla to the sublingual space to identify the etiology. The hypoglossal nerve can be divided into five segments: medullary, cisternal, skull base, carotid space, and sublingual segment [1]. Knowledge of the common pathologies affecting each segment allows the radiologist to provide a concise and relevant differential diagnosis.

MRI and CT play important and complementary roles in evaluation of the hypoglossal nerve pathologies, and optimization of imaging modality and protocolling requires a comprehensive knowledge of regional head and neck anatomy, pathology, and imaging techniques. This article will provide an updated pictorial review of key imaging findings of acute and chronic hypoglossal nerve injury, segmental imaging anatomy of the nerve, and common pathologies which contribute to hypoglossal nerve dysfunction.

## Clinical findings of hypoglossal nerve injury

The hypoglossal nerve is a pure motor nerve, supplying all the intrinsic and extrinsic muscles of the tongue except for the palatoglossus muscle, which is innervated by the pharyngeal plexus, a branch of the vagus nerve with additional contribution from the spinal accessory and possibly glossopharyngeal nerves [2, 3]. Each hypoglossal nucleus receives bilateral input from the primary motor cortex via the corticobulbar tract [4, 5] with the genioglossus receiving crossed innervation [6].

Balanced action of the bilateral genioglossus muscles is necessary to protrude the tongue in the midline without lateral deviation. Thus, hypoglossal palsy may present clinically with dysphagia, dysarthria, or subjective tongue weakness. Examination may reveal unilateral tongue hemiatrophy, deviation, or fasciculations [5]. Unilateral supranuclear lesions rarely cause profound muscle weakness but may cause deviation of the tongue to the contralateral side from the lesion [6]. By contrast, nuclear or infranuclear hypoglossal pathology will lead to tongue deviation toward the side of pathology [4]. Coexistence of other cranial neuropathies may help to further guide clinical and imaging localization of pathology [6].

## Imaging findings of hypoglossal nerve injury

**Fig. 1:**
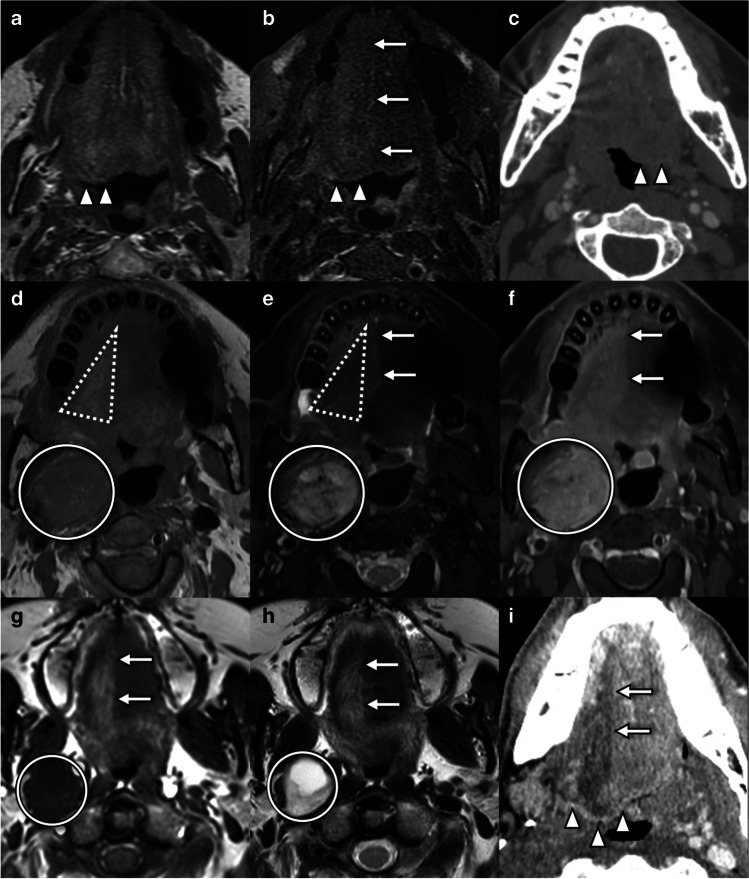
Imaging features of hypoglossal denervation. **a** Axial T1W sequence in a patient with acute tongue ptosis due to cranio-cervical junction degenerative disease (CCJD) (see Fig. 5) demonstrates posterior displacement of the affected right hemitongue (arrowheads), which remains isointense in signal and bulk. **b** Axial T2FS sequence in the same patient as **a** demonstrates posterior displacement (arrowheads) and T2 hyperintensity of the affected right hemitongue. Note the sharply marginated signal change along the median raphe (arrows) which distinguishes this from a mass lesion. **c** Axial contrast-enhanced CT in a 44-year-old patient presenting with neck pain and tongue deviation demonstrates posterior displacement of the left hemitongue (arrowheads) without change in attenuation. A high cervical carotid dissection was identified (not shown). **d** Axial T1W sequence in a 29-year-old patient with chronic tongue ptosis due to a jugular paraganglioma (circle). The affected right hemitongue demonstrates volume loss and T1 shortening (dashed triangle) consistent with fatty atrophy. **e** Axial T2FS sequence in the same patient as **d** demonstrates signal dropout (dashed triangle) within areas of fatty atrophy, but also some areas of high signal attributed to edema. Note how signal changes respect the median raphe (arrows), distinguishing denervation from a mass lesion. In this case, denervation is caused by extrinsic compression by a vagal paraganglioma (circle) in the carotid space, with characteristic T2 hyperintensity and internal flow voids. **f** Axial post-gadolinium T1FS sequence in the same patient as **d** and **e** demonstrates enhancement of the right hemitongue. Note how signal changes respect the median raphe (arrows), distinguishing denervation from a mass lesion. The vagal paraganglioma (circle) enhances markedly. **g** Axial T1W sequence in a 54-year-old patient with tongue weakness and episodic dizziness demonstrates T1 shortening and volume loss in the right hemitongue respecting the median raphe (arrows) compatible with fatty atrophy. In this case, denervation is caused by extrinsic compression by a T1 hypointense schwannoma (circle). **h** Axial T2W sequence in the same patient as **g** demonstrates T2 prolongation and volume loss in the right hemitongue respecting the median raphe (arrows) compatible with fatty atrophy. The causative schwannoma (circle) is of intermediate T2 signal with a T2 hyperintense cystic component. **i** Axial CT in a patient with chronic tongue ptosis due to osseous metastasis (not shown) demonstrates posterior displacement (arrowheads), hypoattenuation, and volume loss in the right hemitongue respecting the median raphe (arrows) compatible with fatty atrophy

Hypoglossal denervation results in characteristic imaging findings (Fig. 1). In the acute and subacute setting, the root of the affected hemitongue may appear enlarged and retracted posteriorly from the oral cavity into the oropharyngeal lumen and should not be mistaken for a mass [7]. Denervation edema, an increase in intramuscular capillary volume and extracellular fluid, manifests as increase in T2 signal intensity and increased gadolinium enhancement in the affected hemitongue [8]. Chronic denervation is characterized by progressive reduction in muscle bulk and increase in fatty infiltration and reflected by ipsilateral volume loss, hypoattenuation on CT, and T1 shortening on MRI [8]. Fibrosis is a rare but described phenomenon [8].

On fluorine-18-fluorodeoxy-d-glucose (FDG) PET/CT, absent tracer uptake may be seen in the denervated hemitongue with increased avidity in the contralateral normal hemitongue. Careful correlation with the site of known disease and scrutiny of the coregistered CT study for hemiatrophy or tongue prolapse is important to avoid misdiagnosis [9].

The geniohyoid, a suprahyoid muscle, and most of the infrahyoid strap muscles are innervated by the ansa hypoglossi, which carries upper cervical spinal root fibers that travel with the hypoglossal nerve in its carotid and sublingual segments, but not fibers of the hypoglossal nerve proper. Thus, if the infrahyoid strap muscles are atrophied along with the ipsilateral tongue muscles, the site of pathology can be localized to the carotid or posterior sublingual space [1].

When hypoglossal palsy is seen with coexistent lower cranial and cervical neuropathies, lesion localization can be further refined. Atrophy of the soft or hard palate, a patulous pyriform sinus, angulated aryepiglottic fold, or paramedian vocal fold are signs of vagal nerve palsy [10], whereas denervation edema or atrophy of the trapezius and sternocleidomastoid indicate involvement of the spinal accessory nerve [11]. These findings should alert the radiologist to potential large lesions in the cisternal segments, skull base, or upper carotid spaces.

Identification of the ptotic tongue or other signs of hypoglossal neuropathy should prompt a thorough assessment of the entire course of the hypoglossal nerve, with segmental localization aiding the differential diagnosis [1]. To do so, one must first thoroughly understand the normal anatomic course of the hypoglossal nerve.

## Normal imaging anatomy

### Nuclei and intramedullary fascicles

The hypoglossal nucleus is located just off-midline in the dorsal medulla, medial to the dorsal vagal nucleus. Each nucleus receives bilateral input from the primary motor cortex [4, 5] via the corticobulbar tract, such that unilateral supranuclear lesions rarely cause profound muscle weakness [6]. Its blood supply is derived from the anterior spinal and vertebral arteries. The inferior aspect of the hypoglossal nucleus bulges along the floor of the fourth ventricle creating the hypoglossal trigone or eminence [12]. The efferent axons of the hypoglossal nerve course ventrally and exit the medulla between the pyramidal tract medially and the inferior olivary nucleus laterally in the lower third of the pre-olivary or ventrolateral sulcus [2].

**Fig. 2:**
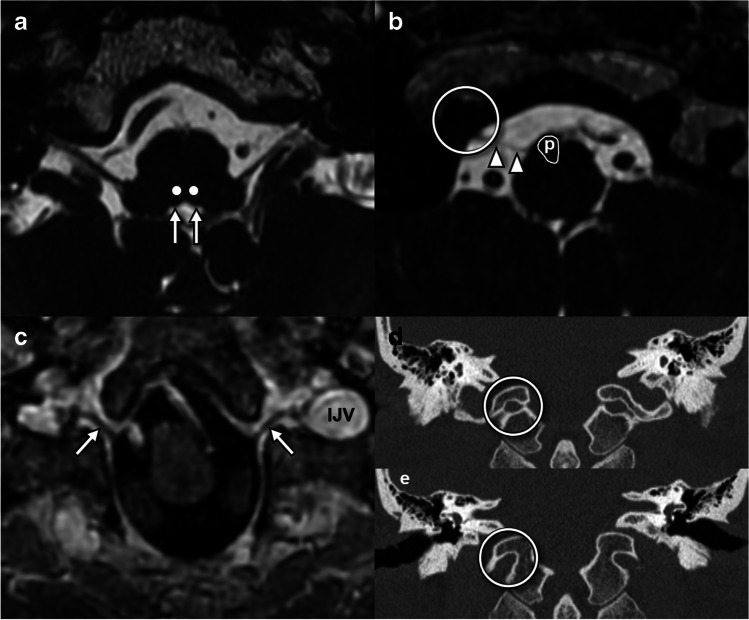
Imaging anatomy of the hypoglossal nuclei and intramedullary fascicles and cisternal and skull base segments. **a** Axial FIESTA sequence through the upper medulla at the level of the inferior olivary nucleus demonstrates the hypoglossal eminence (arrows) at the floor of the fourth ventricle. The expected location of the hypoglossal nuclei (dots) can be inferred from this landmark. **b** Axial FIESTA sequence through the lower medulla below the obex demonstrates HN rootlets (arrowheads) exiting lateral to the pyramidal tracts (p) and into the hypoglossal canal (open circle). **c** Axial post-gadolinium volumetric T1 sequence through the hypoglossal canal demonstrates the canalicular segment of the HN (arrows) as a filling defect surrounded by venous enhancing venous plexus, passing toward the internal jugular vein (IJV) at the upper margin of the carotid sheath. **d** Coronal CT at the level of the internal (posterior) os of the hypoglossal canal where the canal has a circular shape (circle). **e** Coronal CT at the level of the external (anterior) os of the hypoglossal canal with a “bird’s beak” appearance (circle)

In the mature brain, direct visualization of nuclei and fascicles can be difficult, relying on knowledge of anatomic landmarks [13]. The hypoglossal trigone at the floor of the fourth ventricle can be reliably identified on heavily T2W steady-state free precession (SSFP) sequences [14] and to a lesser extent on volumetric FLAIR sequences (Fig. 2a). Visualization on spin-echo T2 and DWI varies by technique depending on slice selection, slice thickness, and intersection gap. However, direct visualization of the fascicular components can be difficult even with SSFP techniques [13].

### Cisternal segment

After exiting the medulla, approximately 12 to 16 rootlets of the hypoglossal nerve merge into 3–6 bundles which travel in the lateral aspect of the pre-medullary cistern, then forming two trunks which pierce the dura just before reaching the hypoglossal canal [14]. Within the pre-medullary cistern, the rootlets pass posterolateral to the vertebral artery and anterior to the posterior inferior cerebellar artery (PICA) [2, 14] where it may have a markedly tortuous course [12].

For the cisternal segment, heavily T2W SSFP sequences allow the nerve rootlets to be directly visualized as linear T2 hypointense structures surrounded by T2 hyperintense cerebrospinal fluid (CSF), originating from the ventrolateral sulcus and traveling within the pre-medullary cistern (Fig. 2b). Time-of-flight MR angiography can be useful in evaluating the relationship between vessels and the components of the hypoglossal nerve. Neurovascular contact with the vertebral artery and PICA is a common finding identified in up to 61% of root bundles amongst healthy volunteers, with distortion of nerve roots in 44% [14] which is not necessarily pathologic.

### Skull base (intracanalicular, foraminal) segment

The trunks of the hypoglossal nerve usually fuse into a single nerve within the hypoglossal canal [12, 14]. The hypoglossal canal is directed antero-infero-laterally through the occipital bone [14] and may be duplicated [4, 12, 15]. The internal os lies superomedial to the jugular foramen, whereas the external os is separated from the jugular foramen by only a thin sheet of bone [16]. Within the canal, the hypoglossal nerve is surrounded by an arachnoid and dural sleeve, occasionally containing CSF, for some two-thirds of the canal [12, 14]. Here, the nerve is surrounded by a venous plexus and accompanied by a meningeal branch of the ascending pharyngeal artery [2, 12].

MRI and CT play complementary roles in the evaluation of the skull base/foraminal segment. Volumetrically acquired gadolinium-enhanced T1 gradient echo sequences are particularly useful for identification of the intracanalicular nerve [14], seen as a slightly tortuous linear filling defect surrounded by enhancing venous plexus [15, 16] (Fig. 2c). Contrast-enhanced SSFP sequences can also reliably delineate the intracanalicular segment [14]. The surrounding normal venous enhancement extends anterolateral to the foramen and into the anterior condylar confluence [16, 17]. The bony hypoglossal canal is best demonstrated on CT (Fig. 2d, e) which is the best modality for assessment of fracture, cortical destruction, or bony remodeling of the hypoglossal canal [4].

### Carotid space (descending) segment

**Fig. 3:**
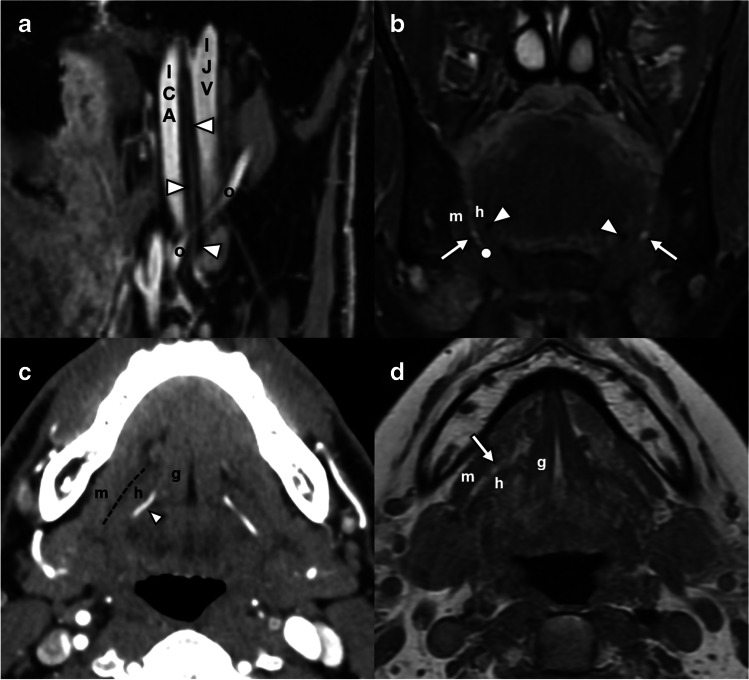
Imaging anatomy of the carotid and sublingual segments of the hypoglossal nerve. **a** Sagittal oblique volumetrically acquired T1FS sequence through the suprahyoid neck demonstrates the path of the HN (arrowheads) in the carotid sheath, between the internal carotid artery (ICA) and the internal jugular vein (IJV) before crossing the occipital artery (o) and turning anteriorly to enter the sublingual space. **b** Coronal T2FS sequence through the floor of mouth at the level of the angle of the mandible demonstrates the anticipated location of the hypoglossal nerve (dot) in the sublingual plane between mylohyoid (m) and hyoglossus (h) muscles and inferior to the submandibular duct (arrow). Note the lingual artery flow voids (arrowheads) deep to hyoglossus. **c** Axial contrast-enhanced CT angiogram demonstrates the plane of the hypoglossal nerve (dashed line) between the genioglossus (g), hyoglossus (h), and mylohyoid (m). Note how the lingual artery (arrowhead) lies deep to hyoglossus. **d** Axial T1W sequence through the floor of mouth demonstrates key landmarks including the genioglossus (g), hyoglossus (h), and mylohyoid (m) and the sublingual fat plane (arrowhead) through which the hypoglossal nerve traverses beneath the submandibular duct

After exiting the hypoglossal canal, the hypoglossal nerve enters the medial aspect of the carotid sheath [4]. Within this space, the nerve descends posteromedial to the internal carotid artery, medial to the internal jugular vein, and in close approximation to the vagus nerve (Fig. 3a). Here, it is joined by somatic efferent fibers from C1 and C2 (ansa hypoglossi) for a short distance, which will eventually supply the motor function of the geniohyoid and infrahyoid strap muscles [4]. The nerve then passes between the internal carotid artery and jugular vein, at which point the superior root of the ansa cervicalis departs [2]. Near the level of the mastoid tip [4], the nerve loops into a horizontal course, crossing superficial to the occipital and external carotid arteries [2, 4] to exit the carotid sheath and enter the sublingual space.

Gadolinium-enhanced MRI yields the best soft tissue contrast in evaluation of the carotid space lesions including carotid dissection, benign and malignant neoplasms, and infectious and inflammatory lesions. However, contrast-enhanced CT remains a fast and relatively inexpensive option, especially in the emergent setting or in patients with contraindications to MRI.

### Sublingual segment

Within the sublingual space, the hypoglossal nerve passes inferior to the posterior belly of the digastric muscle and extends along the lateral surface of the hyoglossus muscle, deep to the mylohyoid sling before continuing anteriorly along the surface of the genioglossus muscle. This segment lies inferior to the sublingual duct and lingual artery [12].

The nerve itself is not easily visualized in this segment with conventional MR or CT. However, muscular, vascular, and ductal landmarks can be readily identified to infer the course of the hypoglossal nerve (Fig. 3b, c, d). Conversely, CT sensitivity for lesion detection may be limited by beam hardening and photon starvation artifacts from the adjacent mandible and dentition, which may be exacerbated if there is metallic dental hardware.

## Optimizing imaging of the hypoglossal nerve

Imaging the hypoglossal nerve in cases of tongue ptosis and atrophy is essential, as clinical examination alone cannot localize the site of injury. On all modalities, the hypoglossal nerve must be imaged through its entire course not only to localize the primary lesion but also to identify potential perineural spread of tumor in the setting of head and neck malignancy. Intravenous contrast is essential for detection of small tumors, diagnosis of perineural malignancy, and characterization of infectious or inflammatory lesions.

CT and MRI have complementary roles in imaging the hypoglossal nerve. CT facilitates evaluation of the hypoglossal canal and skull base and characterization of osseous lesions. While the nerve itself is not directly visualized on CT, the use of intravenous contrast can allow for delineation of vascular relationships to a known lesion, or better characterize the enhancement characteristics of a compressive lesion.

MRI allows for direct visualization of nerve segments and is commonly the method of choice for imaging a patient presenting with hypoglossal nerve palsy. An MRI protocol for imaging the hypoglossal nerve follows the general principals of imaging other cranial nerves and cranial nerve pathology. In the cisternal segment, the nerve is best evaluated with heavily T2W SSFP sequences. Time-of-flight MR angiography can be useful in evaluating vascular relationships intracranially. For the foraminal and extracranial segments, high-resolution T1W sequences without and with gadolinium enhancement provide the best visualization and assessment for possible perineural spread of malignancy, infection or inflammation, and small lesions. Of note, pre-contrast T1W sequences should be performed without fat suppression, in order to take advantage of the intrinsic contrast offered by T1 hyperintense soft tissue fat and bone marrow against potential lesions, which tend to demonstrate relative T1 signal hypointensity. Postcontrast T1W imaging can be performed with or without fat suppression, with fat-suppressed images offering higher conspicuity of enhancing lesions but potentially suffering more from susceptibility and motion artifacts. In general, high-contrast signal-to-noise ratio of 2D spin-echo sequences allows optimal evaluation of fat planes, whereas 3D-T1 gradient sequences are useful for their high spatial resolution and relative speed. Compared to gradient-based sequences, 3D turbo spin-echo sequences, both T1 and T2 weighted, offer intrinsically lower susceptibility artifact and has demonstrated promise in skull base and cranial nerve imaging, both intracranially and extracranially [18–20]. Some authors also advocate for use of balanced SSFP sequences with and without gadolinium to evaluate the extraforaminal cranial nerves, as the enhancement of surrounding structures, such as venous plexuses or pathology, better distinguishes them from the intrinsically hypointense, non-enhancing cranial nerve [13].

## Common pathology

### Nuclei and intramedullary fascicles

Neoplasms are the most common pathology affecting the hypoglossal nucleus and intramedullary fascicles. Pathology at this level is commonly associated with complex lower cranial neuropathy resulting from injury to multiple cranial nerve nuclei. Due to their relatively midline location, it is not uncommon for both hypoglossal nuclei to be affected a lesion at this level, resulting in complete tongue paralysis.

**Fig. 4:**
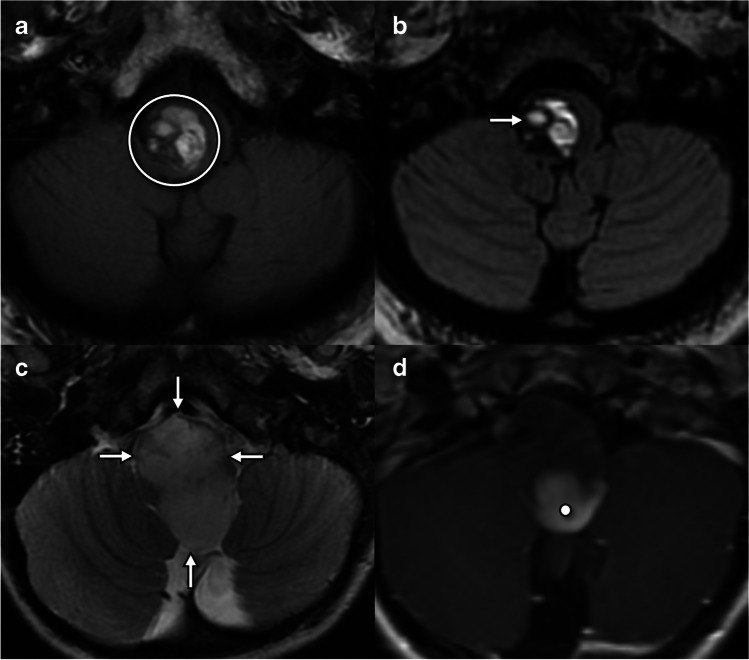
Lesions involving the hypoglossal nuclei and intramedullary fascicles. **a** Axial T1W through the lower medulla in a 56-year-old presenting with headaches, nausea, and tongue weakness demonstrates a multilobulated lesion with intrinsic T1 shortening compatible with subacute blood products (circle). **b** Axial T2W in the same patient as **a** shows the multilobulated lesion contents are mostly T2 hyperintense with “shading” (arrow) suggesting blood products of varying ages and a peripheral T2 hypointense rim, typical of a cavernous venous malformation. **c** Axial T2W in a 4-year-old with headaches and lower cranial neuropathies demonstrates an expansile T2 hyperintense mass (arrows) occupying the medulla. **d** Axial T1W+C in the same patient as **c** demonstrates solid nodular enhancement at the periphery of the mass (dot). No reduced diffusion (not shown). Operative pathology confirmed pilocytic astrocytoma (WHO CNS grade 1)

Any pathology which occurs in the medulla can potentially contribute to hypoglossal dysfunction (Fig. 4). Neoplastic considerations include both primary central nervous system (CNS) tumors such as glioma, the most common primary medullary neoplasm, and secondary parenchymal metastatic disease. Medullary infarct and hemorrhage can cause acute onset hypoglossal dysfunction. Infectious and inflammatory conditions, including demyelinating diseases such as multiple sclerosis, may also contribute to hypoglossal pathology.

### Cisternal segment

**Fig. 5:**
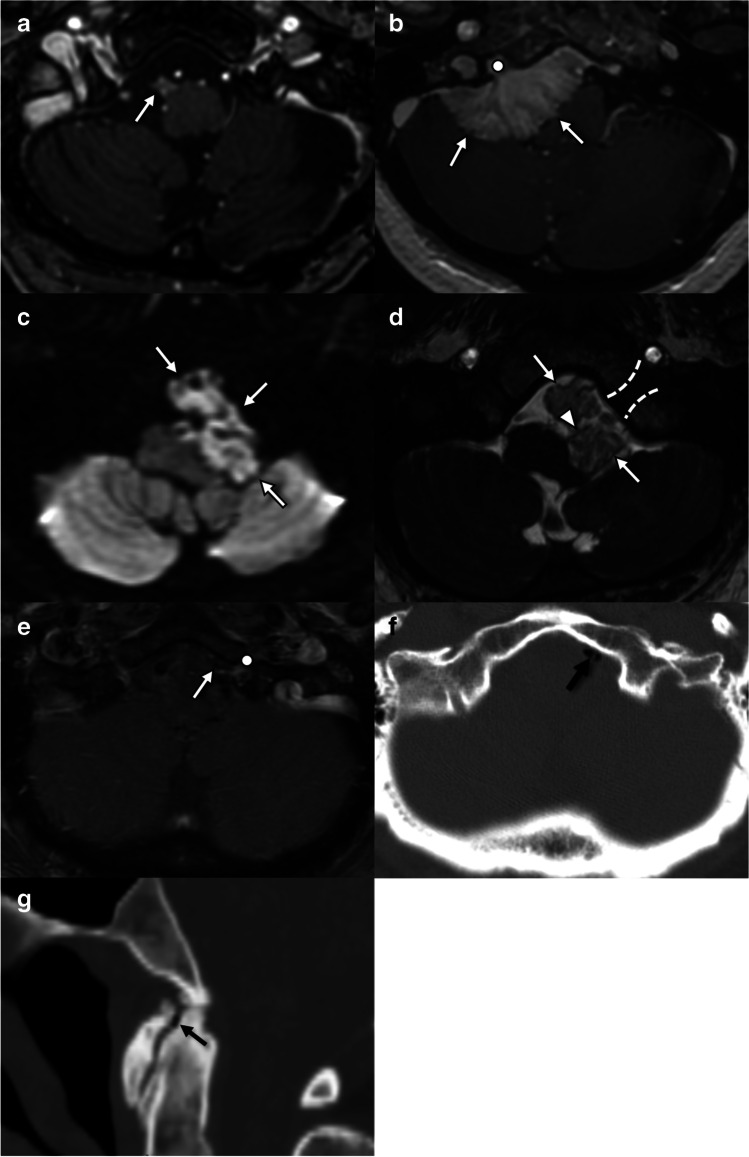
Lesions involving the cisternal segment of the hypoglossal nerve. **a** Axial post-gadolinium T1W SPGR sequence in a 44-year-old with history of NF2 and prior left vestibular schwannoma resection demonstrates an enhancing nodule arising from the right lateral medulla from a hypoglossal nerve root. **b** Axial post-gadolinium T1W SPGR sequence in a 66-year-old with multiple right-sided cranial nerve palsies demonstrates a homogenously enhancing lobulated dural-based mass (arrow) arising from the right petrous face and extending into the hypoglossal canal (dot). Surgical pathology confirmed WHO grade 1 meningioma. **c** Axial DWI (*b* = 1000) in a 14-year-old female with headache and slowly progressive lower cranial neuropathies demonstrates a lobulated extra-axial cystic lesion in the left cerebellomedullary cistern with markedly reduced diffusion (arrows) consistent with an epidermoid cyst. **d** Axial FIESTA in the same patient as **c** clearly shows left hypoglossal nerve rootlets (arrowhead) traversing the epidermoid cyst (arrows) en route to the hypoglossal canal (dashed lines). **e** Axial post-gadolinium T1W in a 54-year-old with chronic left-sided tongue weakness is notable for a non-enhancing T1 hypointense extradural lesion (arrow) at the orifice of the left hypoglossal canal (dot). This was of low signal intensity on T2W (not shown). **f** Axial unenhanced CT in the same patient as **e** reveals the lesion is of gas density (black arrow). **g** Sagittal reformatted unenhanced CT image of the midline skull base in the same patient as **e** demonstrates marked degenerative changes at the cranio-cervical junction with intra-articular gas (black arrow). This was thought to have migrated to the extradural space in **f** and resulted in cisternal impingement of the hypoglossal nerve

The cisternal segment of the hypoglossal nerve is often affected by pathology extending from the skull base, usually neoplastic lesions such as meningiomas or chordomas (Fig. 5). Nerve sheath tumors or leptomeningeal spread of malignancy may also affect hypoglossal rootlets in the cerebellomedullary cistern. Extra-axial cystic lesions including arachnoid, epidermoid, or neurenteric cysts may distort the nerve due to mass effect. Juxta-articular synovial cysts, contiguous with and projecting superiorly from a cranio-cervical junction synovial joint, may also result in compressive neuropathy [21]. High-resolution T2W SSFP sequences allow best visualization of the nerve roots in relation of the vascular structures in these cases.

### Skull base (intracanalicular) segment

**Fig. 6:**
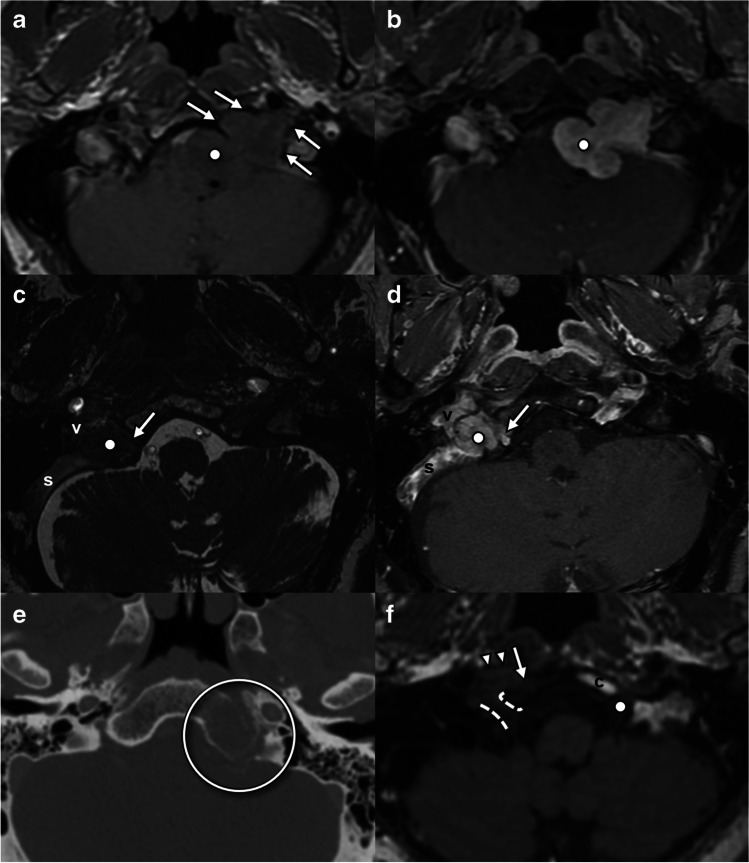
Lesions involving the intracanalicular segment of the hypoglossal nerve. **a** Axial T1W in a 44-year-old patient with chronic left-sided tongue weakness demonstrates a dumbbell-shaped mass (dot) extending from the left cerebellomedullary cistern causing smooth remodeling of the hypoglossal canal (arrows). **b** Axial post-gadolinium T1W in the same patient as **a** confirms marked, homogenous enhancement of the mass (dot), subsequently proven to be a schwannoma by fine needle aspiration. **c** Axial SSFP in a 55-year-old male with multiple right lower cranial neuropathies demonstrates a mass (dot) occupying the right jugular bulb, with involvement of the right hypoglossal nerve (arrow) in within the hypoglossal canal and with partial occlusion of the sigmoid sinus (s) and the internal jugular vein (v). **d** Axial post-gadolinium T1W in the same patient as **c** confirms enhancement of the mass, which was later biopsy proven to be a jugular paraganglioma. **e** Axial CT bone reconstructions following intravenous iodinated contrast in a patient with history of metastatic NSCLC demonstrates permeative erosion of the left basi-occiput involving the left hypoglossal canal (circle) due to a skull base metastasis. **f** Axial T1W in a 66-year-old patient with metastatic NSCLC demonstrates hypointense T1 signal in the right clivus and cortical destruction (arrow) due to a metastatic deposit with extraosseous extension (arrowheads). T1 hypointense mass involves the right hypoglossal canal (dashed lines). The normal left hypoglossal canal (dot) is hyperintense on T1W and surrounded by T1 hyperintense clival fatty marrow (c)

Tumors both benign and malignant are the most common cause of nerve damage at this level (Fig. 6). Tumors may extend from the skull base itself resulting in erosion and destruction of the hypoglossal canal within the occipital bone. The most common malignant tumors are metastases from breast, lung, and prostate primary malignancies. Nasopharyngeal carcinoma may extend directly through the skull base affecting the hypoglossal nerve.

Benign tumors that commonly affect the nerve at this location include peripheral nerve sheath tumors, glomus tumors (glomus jugulare), and meningiomas [21]. Central or nodular peripheral enhancement is typical of peripheral nerve sheath tumors such as schwannomas [21], whereas the enhancement pattern is more variable in neurofibromas [22]. Paragangliomas of the jugular foramen (glomus jugulare) may affect the nerve due to the close relationship of the hypoglossal canal and jugular foramen. Dural AV fistulas may involve the hypoglossal canal and can result in hypoglossal palsy [17]. Hypoglossal canal non-enhancing cysts, including nerve root sleeve meningeal cysts, may widen the hypoglossal canal but are not usually associated with denervation changes [21].

### Carotid space segment

**Fig. 7:**
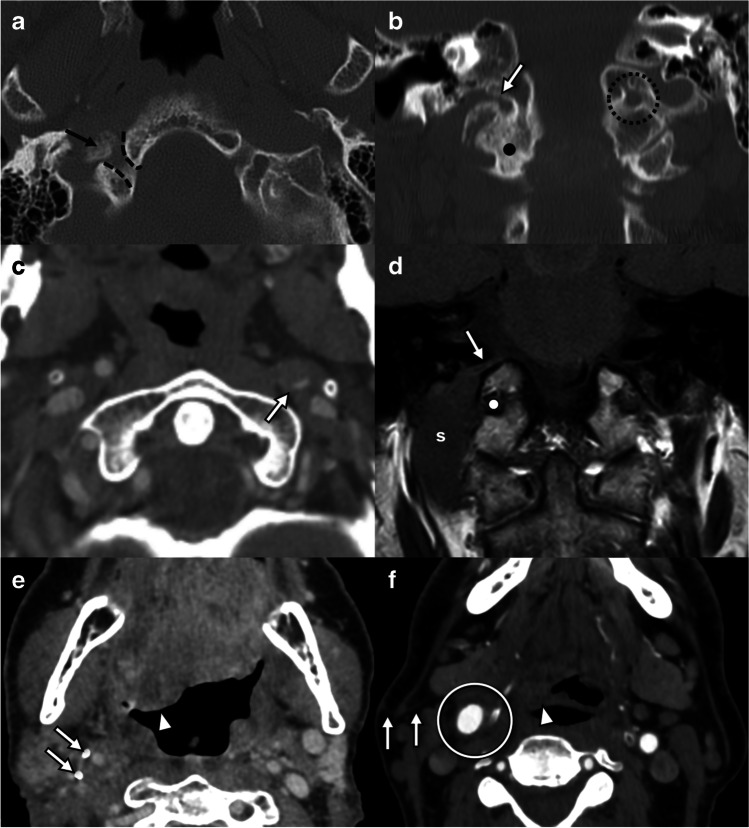
Lesions involving the carotid segment of the hypoglossal nerve. Further tumors of the carotid sheath have been presented in Fig. 1. **a** Axial unenhanced CT in a 76-year-old female with prior cervical fusion surgery presents with right tongue weakness after trigger point injections reveals an osteophyte (arrow) overlying the external os of the right hypoglossal canal (dashed line). **b** Coronal unenhanced CT in the same patient as **a** reveals ankylosis of the right C1-C2 articulation contiguous with the osteophyte (dot) causing stenosis of the right hypoglossal canal (arrow). The left hypoglossal canal (circle) is normal in caliber. **c** Axial contrast-enhanced CT angiogram in a 44-year-old presenting with neck pain and tongue weakness demonstrates irregular narrowing of the left cervical internal carotid artery lumen consistent with a dissection flap (arrow). Left tongue ptosis was shown in Fig. 1c. **d** Coronal T1W in the same patient as Fig. 1f, a 54-year-old patient with tongue weakness and episodic dizziness, demonstrates a fusiform T1 hypointense schwannoma (s) arising from pars nervosa of the right jugular foramen (arrow) and causing extrinsic mass effect on the right hypoglossal canal (dot). Further tumors of the carotid sheath have been presented in Fig. 1. **e** Axial contrast-enhanced CT neck in a 62-year-old 3 months after salvage right neck dissection for nodal recurrence of oropharyngeal SCC demonstrates surgical clips in the right carotid space (arrows) and effaced suprahyoid fat planes due to post-treatment changes as well as acute right hypoglossal denervation (arrowhead). There was no evidence of perineural disease on MRI (not shown). **f** Axial contrast-enhanced CT angiogram of the neck in a 61-year-old 1 month after right carotid endarterectomy demonstrates linear scarring (arrows) and loss of fat planes (circle) in the right carotid sheath due to post-surgical changes and acute right hypoglossal denervation (arrowhead). Follow-up CT angiogram for planning of contralateral endarterectomy demonstrated resolution of the right hypoglossal palsy (not shown)

Carotid endarterectomy is a common iatrogenic cause of hypoglossal palsy [5] due to either inadvertent trauma or ischemic injury [2] (Fig. 7). Other vascular etiologies include carotid artery dissection, aneurysm and pseudoaneurysm, and jugular vein thrombosis [22]. Malignancy involving the carotid space is most frequently extranodal extension of metastatic adenopathy from a primary carcinoma of the skin or aerodigestive tract [22]. Furthermore, the hypoglossal nerve may be involved by direct invasion or perineural spread of a primary lesion including squamous cell carcinoma, lymphoma, salivary gland malignancy, or soft tissue sarcomas. Perineural tumor manifests as asymmetric thickening or enhancement of the nerve on post-gadoliniuim T1W fat-saturated sequences. Benign peripheral nerve sheath tumors may also result in compressive neuropathy as described above [22].

### Sublingual segment

**Fig. 8:**
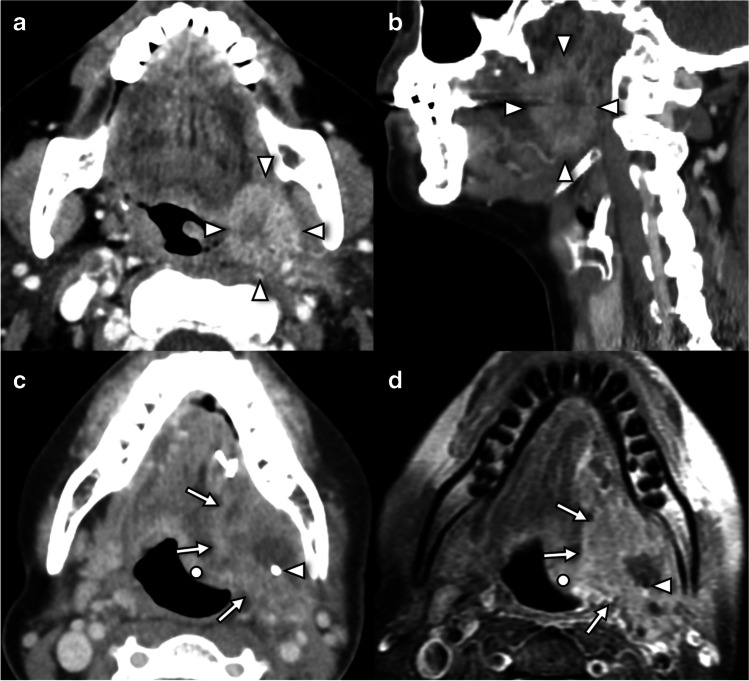
Lesions involving the sublingual segment of the hypoglossal nerve. **a** Axial contrast-enhanced CT in a 61-year-old female smoker with left neck pain and swallowing difficulty reveals a centrally necrotic, enhancing and infiltrative mass in the left glossotonsillar sulcus (arrowheads) consistent with squamous cell carcinoma. **b** Sagittal contrast-enhanced CT in the same patient as **a** demonstrates the mass (arrowheads) occupies the expected location of the hypoglossal nerve at the junction of carotid and sublingual space segments. **c** Axial contrast-enhanced CT in a 65-year-old male smoker status post partial glossectomy, neck dissection, and radiotherapy for left oral tongue SCC demonstrates a bulky, necrotic recurrent mass (arrows) around surgical clips (arrowhead) and accompanying left tongue ptosis (dot). **d** Axial contrast-enhanced T1W in the same patient as **c** better demonstrates extent of soft tissue involvement by recurrent tumor (arrows) around the surgical resection clips (arrowhead) with associated denervation ptosis (dot)

The most common pathology affecting the hypoglossal nerve at this level is related to squamous cell carcinoma of the base or lateral oral tongue [1] (Fig. 8). Obliteration of the normal fat planes adjacent to the proximal aspect of the lingual artery is an important sign of potential hypoglossal nerve involvement.

Tongue dysfunction post-operatively following resection of a primary head and neck malignancy or neck dissection is indicative of iatrogenic injury to the nerve during surgery. If the nodal dissection or primary tumor extends to involve the submandibular triangle, there is an increased incidence of nerve damage following surgery. New hypoglossal denervation during surveillance imaging should prompt careful scrutiny for recurrent or perineural disease.

### Lesions without ptosis

**Fig. 9:**
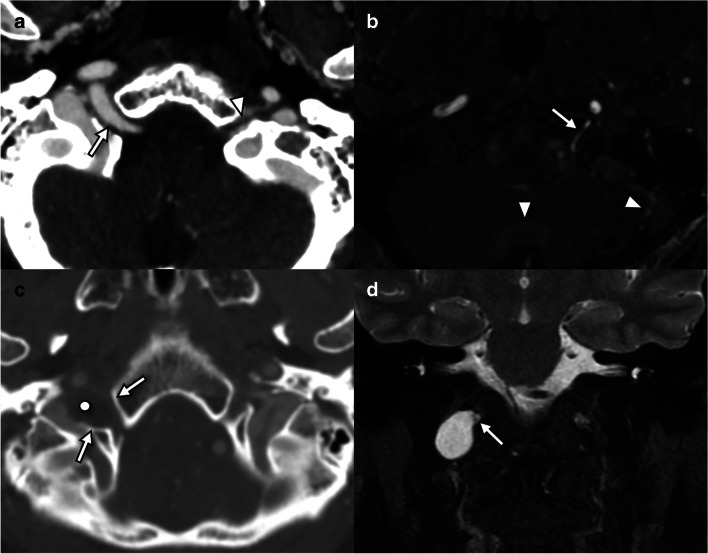
Lesions of the hypoglossal canal which are not commonly associated with denervation. **a** Axial CT angiography in an adult patient presenting with transient ischemic attack demonstrates an artery coursing through the right hypoglossal canal (arrow) forming an anastomosis between the right internal carotid artery and the basilar trunk (not shown), a persistent primitive hypoglossal artery, and a congenital anomaly. Note the normal left hypoglossal nerve (arrowhead), seen as a filling defect surrounded by venous plexus. This patient did not have hypoglossal palsy. **b** Axial time-of-flight MR angiogram in a 73-year-old (male) with pulsatile tinnitus demonstrates a high flow vessel traversing the left hypoglossal canal (arrow) and with arterialized flow in the dural venous sinuses (arrowheads). DSA (not shown) revealed multiple dural arteriovenous fistulas. This patient did not have hypoglossal palsy. **c** Axial CT angiography in a 59-year-old (female) presenting with an episode of dizziness reveals smooth remodeling of the right hypoglossal canal (arrows) due to a hypoattenuating cystic lesion (dot). **d** Coronal T2W MRI in the same patient as **c** demonstrates a T2 hyperintense circumscribed lesion protruding through the right hypoglossal canal (arrow), consistent with perineural sleeve cyst. It was isointense to CSF on all sequences and did not demonstrate any enhancement or reduced diffusion (not shown). This patient did not have hypoglossal palsy

Several imaging findings localize to the course of the hypoglossal canal but are rarely associated with palsy (Fig. 9). The hypoglossal artery represents a persistent fetal connection between the origin of the cervical internal carotid artery and the contralateral vertebral artery or basilar artery [23]. In persistent hypoglossal artery, the ipsilateral vertebral artery and cervical foramina transversaria are absent, and the hypoglossal canal is enlarged [23]. Dural arteriovenous fistula may also manifest with a dilated artery traversing the hypoglossal canal, but only a small subset results in hypoglossal palsy [17]. Non-enhancing cystic lesions of the hypoglossal canal, including meningeal sleeve cysts of the hypoglossal nerve, do not result in hypoglossal palsy [21].

## Conclusion

When faced with imaging findings of unilateral tongue ptosis, comprehensive knowledge of hypoglossal nerve anatomy and its surrounding structures is essential to localize and diagnose potential pathology. This article will help radiologists review the complex nerve anatomy in a segmental approach and provide imaging examples of diverse pathology that may contribute to tongue ptosis.

## Abbreviations

CCJD: Cranio-cervical junction degenerative disease
CNS: Central nervous system
CSF: Cerebrospinal fluid
CT: Computed tomography
FDG: Fluorine-18-fluorodeoxy-d-glucose
MRI: Magnetic resonance imaging
SSFP: Steady-state free precession
T1W: T1-weighted
T2W: T2-weighted
